# The American Society of Clinical Oncology Reports on the State of Cancer Care in America: 2017

**Published:** 2017-07-01

**Authors:** Pamela Hallquist Viale

The American Society of Clinical Oncology (ASCO) has recently published its State of Cancer Care in America report for the year 2017. This is a yearly compilation of the trends and issues facing cancer care in America, giving readers a thorough look at important challenges and improvements in the care of patients with cancer (ASCO, 2017).

## TRANSFORMATIONS

ASCO reports that our healthcare system is being transformed to improve the management of patients with cancer. The transformation is centered on three basic pillars supporting patient care needs: new investments in science and insurance coverage, new payment systems that emphasize quality, and new sources of data and rapid-learning health-care systems. The report also emphasizes that reduced rates of incidence and mortality for many common cancers continue, yet increased numbers of survivors coupled with more diagnosed patients challenge patient access to appropriate and fiscally responsible care (ASCO, 2017). In 2016, five new drugs and biologic therapies were approved and added to our armamentarium of over 200 approved antineoplastic agents. Expanded use of older agents and new diagnostics also added to the improved management of patients with cancer.

## MOONSHOT INITIATIVE

The Cancer Moonshot initiative spearheaded by President Obama and Vice President Biden heightened interest in cancer and research by focusing on eligibility to clinical trials, brokering financial commitments from private enterprises, and broadening access to clinical trial information for patients (ASCO, 2017). With an increasing population in America, the number of new cancer diagnoses and survivors continues to increase. Some specific tumor types remain difficult to treat, including pancreatic, brain, and lung tumors. The Affordable Care Act (ACA) increased the number of insured patients, helping to reduce the barriers to accessible and affordable care and demonstrating improved outcomes (ASCO, 2017).

## PRACTICE AND WORKFORCE TRENDS

The report detailed changes in oncology practice and workforce trends, noting that practices are facing a rapidly changing landscape coupled with ongoing pressures in practice. The annual Oncology Practice Census survey and Workforce Information System stated that oncology practices are straining under increased practice expenses. The report noted that a published study attributed 49% of physician time to electronic health records vs. 27% to patient care; another study demonstrated vast numbers spent on quality reporting (Casalino et al., 2016; Sinsky et al., 2016). The report commented on the workforce, noting that 12,100 oncology specialists cared for patients in the year 2016, but also that the population of specialists is aging, with 1 in 5 oncologists approaching the age of 64 or older.

The Oncology Practice Census survey represented 1,581 sites of care across the country, 8,400 oncology specialists, 25,000 associated physicians (from other specialties), and 4,100 oncology-specific advanced practice providers (APPs; ASCO, 2017).

Uneven geographic distribution of physicians remains a problem, especially in rural areas. The use of telemedicine has been reported as a means to help meet this challenge. Changes in the responsibilities of cancer care providers require professionals to recast their roles within their clinics and beyond (ASCO, 2017). Challenges in the move toward high-quality and value-based cancer care require systems to address the rising costs of care. Anticipated changes from the implementation of the Medicare Access and Children’s Health Insurance Program (CHIP) Reauthorization Act of 2015 (MACRA) were detailed. Other players in high-quality and value-based care include the Oncology Care Model (OCM) and the ASCO Patient-Centered Oncology Payment (PCOP) model.

The affordability of cancer care continues to be a significant issue. Although the ACA increased the number of insured, even insured patients can suffer financially because of their disease. This financial toxicity can cause emotional and physical distress and impact quality of life. With rising costs, insurers have shifted some of the financial burden to patients with cancer, leading to high deductibles and higher drug copays (ASCO, 2017). Costs for oncology drugs have risen precipitously, with patients often sharing larger amounts of the cost.

## ADVANCED PRACTICE PROVIDERS

I am pleased that the report acknowledged the expanded use of APPs and support for our roles and contributions. Oncology practices were asked to provide perspectives on the roles and availability of APPs. The majority of respondents noted that nurse practitioners and physician assistants were instrumental in the ordering and administering of chemotherapy, symptom and pain management, as well as providing primary care to patients with cancer and survivors. Of the 443 practices that responded to the Oncology Practice Census survey, 75% reported employment of APPs (ASCO, 2017). One in three of the practices reported hiring more APPs, with 6% noting decreased numbers of the use of APPs. A new survey specifically examining the contributions and roles of APPs will be published later this year.

## FUTURE DISCUSSIONS

I’ve given you some highlights of the ASCO report, but I urge you to read it in full. Access it here: https://doi.org/10.1200/JOP.2016.020743. For those of us working in oncology today, the report gives a comprehensive look at the critical issues we face. We can anticipate further discussion of our roles and contributions when the new ASCO survey on APPs is published. With all of the changes occurring or anticipated in oncology care, APPs need to stay informed and engaged to maintain a place at the table for decisions in care.
